# Supplementary figures and images for: A modified BCG with depletion of enzymes associated with peptidoglycan amidation induces enhanced protection against tuberculosis in mice
Source: eLife. 2024 Apr 19;13:e89157. doi: 10.7554/eLife.89157 (PMC11132681; doi:10.7554/eLife.89157)

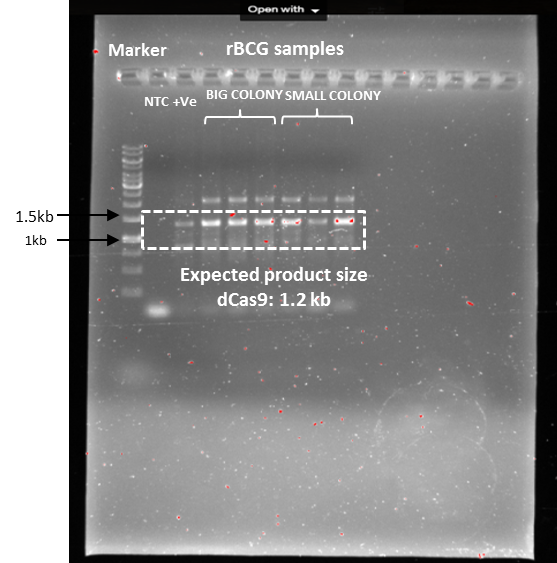

Supplement: Figure 2—figure supplement 2—source data 1. [file elife-89157-fig2-figsupp2-data1.zip › Figure 2- figure supplement 2d source/Figure 2-figure supplement 2d source relevant bands labelled.tif]

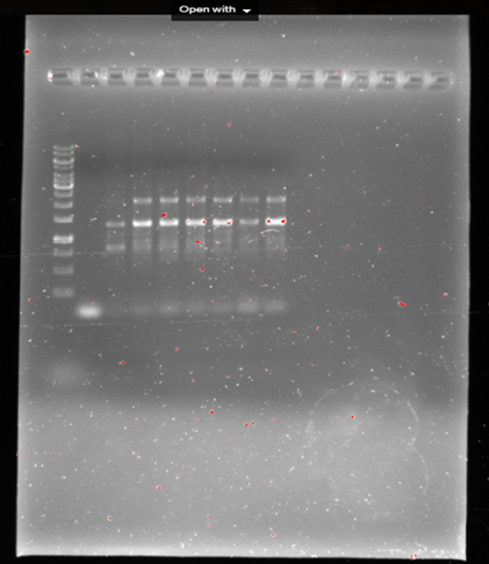

Supplement: Figure 2—figure supplement 2—source data 1. [file elife-89157-fig2-figsupp2-data1.zip › Figure 2- figure supplement 2d source/Figure 2-figure supplement 2d source raw unedited.tif]
